# Salivary Calculus

**Published:** 1856-01

**Authors:** 


*Salivary Calculus.*—Dr. Davis, of Baltimore, presented to the senior editor, a few weeks since, a concretion of salivary calculus, about one and a half inches long and about five-eighths in diameter, of a cylindrical shape, and as compact in its structure almost as ivory. It was taken from the parotid duct of a horse. We intend to have a portion of it analyzed, the result of which, we will give in a future number of the Journal.

